# RpaA Overexpression Enhances Bioluminescence Intensity and Elevates Rhythmic Extracellular Vesicle Yield in *Synechococcus elongatus* PCC 7942

**DOI:** 10.3390/life16060885

**Published:** 2026-05-25

**Authors:** Xiaoshuang Liu, Wenlei Zhang, Lu Wang, Ronggui Li, Chi Zhao, Xuefeng Lu

**Affiliations:** 1 College of Life Sciences, Qingdao University, Qingdao 266071, China; liuxs@qibebt.ac.cn; 2 Key Laboratory of Photoelectric Conversion and Utilization of Solar Energy, Qingdao Institute of Bioenergy and Bioprocess Technology, Chinese Academy of Sciences, Qingdao 266101, China; 3 Shandong Energy Institute, Qingdao 266101, China; 4 Qingdao New Energy Shandong Laboratory, Qingdao 266101, China; 5 Laboratory for Marine Biology and Biotechnology, Qingdao Marine Science and Technology Center, Qingdao 266237, China

**Keywords:** cyanobacteria, circadian rhythm, synthetic biology, metabolic engineering

## Abstract

The cyanobacterial circadian clock orchestrates a wide range of physiological processes. However, its involvement in extracellular vesicle (EV) biogenesis remains largely unexplored. In this study, we investigated the role of the master circadian output regulator RpaA in modulating EV production in *Synechococcus elongatus* PCC 7942. Deletion of *rpaA* simultaneously abolished the bioluminescence rhythmicity and rhythmic EV production, whereas overexpression of *rpaA* enhances the output of circadian clock signals in a dose-dependent manner and markedly increases the bioluminescence intensity and EV production while maintaining the normal circadian oscillation patterns. Transcriptional analyses indicated that *rpaA* positively regulates *lpxD* to promote membrane synthesis and differentially inhibits *glgC* transcription to optimize carbon allocation. These findings reveal a previously unrecognized link between circadian output signaling and EV biogenesis and suggest that *rpaA* overexpression is an effective strategy for improving the yield of EVs. It also provides novel insights and regulatory targets for facilitating the synthesis of other high-value metabolites in cyanobacterial cell factories, and holds certain practical significance for advancing cyanobacterial synthetic biology research and the application of nanocarrier technology.

## 1. Introduction

Cyanobacteria are photosynthetic prokaryotes that have profoundly shaped the Earth’s ecosystem since their emergence over 3 billion years ago, primarily through their pioneering role in oxygenic photosynthesis [1]. These organisms function as major contributors to global carbon and nitrogen cycling, serving as primary producers in diverse aquatic and terrestrial environments [2]. Beyond their ecological significance, cyanobacteria have emerged as promising chassis organisms for synthetic biology applications due to their genetic tractability, efficient CO_2_ fixation capabilities, and potential for sustainable bioproduction of fuels, chemicals, and pharmaceuticals without requiring organic carbon sources [3,4,5]. Among model cyanobacteria, *Synechococcus elongatus* PCC 7942 stands out as a particularly well-characterized system, offering powerful genetic tools for fundamental research and biotechnological applications [6,7].

The circadian clock system of *Synechococcus elongatus* PCC 7942 represents the simplest known circadian mechanism, consisting of three core oscillator proteins KaiA, KaiB, and KaiC, which generate autonomous 24 h rhythms of KaiC phosphorylation in vitro [8]. This remarkable post-translational oscillator integrates environmental timing cues through metabolic signals and transmits temporal information via the SasA-RpaA two-component system [9]. RpaA functions as a master transcriptional regulator controlling global gene expression patterns, directly binding to over 170 gene promoters and orchestrating a sigma factor cascade involving RpoD2, RpoD6, RpoD5, and SigF2 that generates complex circadian expression programs [10,11]. RpaA’s phosphorylation state oscillates rhythmically, activating gene expression during subjective day, while its paralog RpaB mediates transcriptional repression during subjective night through competitive binding to clock-regulated promoters [12]. This sophisticated regulatory network enables cyanobacteria to anticipate daily environmental changes and temporally separate incompatible metabolic processes, conferring significant fitness advantages under rhythmic light–dark cycles [13,14]. RpaA plays an essential role in maintaining circadian rhythms in cyanobacteria. Previous studies have demonstrated that knocking out the *rpaA* gene leads to either complete arrhythmia or attenuated oscillation. Furthermore, the *rpaA*-KO strain exhibits comparable growth rates to the wild-type (WT) strain under constant light (LL) conditions, whereas it displays impaired growth under light–dark cyclic (LD) conditions [9]. Nevertheless, recent studies suggest that supplementing with 3% CO_2_ effectively rescues this growth defect in LD conditions. This recovery is driven by the alleviation of carbon-scarcity-induced redox stress, which is the principal cause of nighttime ROS toxicity in circadian mutants. The finding that an enriched CO_2_ environment obviates the need for a functional circadian clock suggests that this oscillatory system may have emerged as an evolutionary adaptation to the declining atmospheric CO_2_ levels that followed the Great Oxidation Event [15].

Extracellular vesicles (EVs) represent an underappreciated secretion system in cyanobacteria, comprising nanoscale bilayered membrane structures (20~400 nm) that bud from the outer membrane and contain diverse cargo, including proteins, lipids, nucleic acids, and metabolites [16,17]. While EV biogenesis mechanisms remain incompletely understood in cyanobacteria, these EVs appear to constitute regulated secretion processes rather than accidental membrane blebbing events [18]. Cyanobacterial EVs have been observed in multiple species, including *Synechococcus elongatus* PCC 7942, *Synechocystis* sp. PCC 6803, *Prochlorococcus*, and *Leptolyngbya boryana*, where they mediate various physiological functions such as stress responses, metal detoxification, and intercellular communication [19,20,21]. Notably, preliminary evidence suggests that EV formation may exhibit temporal patterns, though the systematic characterization of circadian regulation of vesiculation in cyanobacteria remains unexplored. In *Synechococcus elongatus* PCC 7942, the circadian clock optimizes fitness by partitioning carbon resources between daytime accumulation and nighttime utilization. Two key genes involved in this metabolic orchestration are *glgC* and *lpxD*. *glgC* encodes ADP-glucose pyrophosphorylase, the rate-limiting enzyme for glycogen synthesis, while *lpxD* is involved in the biosynthesis of lipopolysaccharides (LPSs), a primary component of the outer membrane from which EVs originate. Both genes exhibit robust circadian rhythms [10,22]. Analyzing these genes offers insight into how RpaA shifts carbon flux from storage to membrane synthesis, thereby supplying the structural precursors required for rhythmic EV biogenesis.

The biomedical potential of cyanobacterial EVs is also gaining recognition, with recent studies demonstrating promising therapeutic applications. *Spirulina*-derived EVs have shown pro-inflammatory and immune-stimulatory properties, making them potential vaccine adjuvants [23]. *Synechocystis* EVs have been successfully engineered as protein carriers for zebrafish, demonstrating accumulation in gastrointestinal tissues following immersion treatment [24]. Additionally, *Synechococcus elongatus* PCC 7942 EVs promote wound healing through interleukin-6 expression [25]. These findings highlight cyanobacterial EVs as safe, biocompatible and low-immunogenicity nanocarriers for therapeutic applications.

Manipulating circadian clock components has emerged as a strategy to enhance cyanobacterial bioproduction yields. Previous studies have demonstrated that either overexpressing the core clock component *kaiA* [26] or deleting the negative circadian output regulator *labA* [27] can substantially increase the yields of heterologous proteins in *Synechococcus elongatus* PCC 7942. Furthermore, strains with circadian periods matching environmental light–dark cycles demonstrate superior competitive fitness through “resonance” effects [13], while clock mutations can drive adaptive plasticity, enabling rapid growth under specific conditions [28]. Although the feasibility of enhancing EV yield via circadian clock manipulation, particularly through the modulation of the RpaA-mediated regulatory pathways, remains poorly elucidated, RpaA represents a promising alternative target for the improvement of bioproduct yields. This inference is supported by previous findings that the bioluminescence amplitude of *rpaA*-KO is significantly reduced relative to wild-type (WT) [9]. The abundance of the RpaA protein is presumed to be closely correlated with bioproduct accumulation.

Despite the growing interest in both cyanobacterial circadian regulation and EV biology, the interconnection between these two fields remains poorly understood. Specifically, it is currently unknown whether vesiculation exhibits circadian rhythmicity and how the core clock component RpaA, which is the master transcriptional regulator, mediates membrane trafficking and EV formation. Furthermore, while circadian clock manipulation has proven effective for metabolic engineering, its potential to enhance EV yields has not yet been explored. This study bridges these gaps by identifying novel downstream processes regulated by the circadian oscillator, thereby establishing a fundamental link between the cellular timekeeping system and EV-biogenic pathways.

The objectives of this study are to elucidate the role of RpaA in modulating bioluminescence intensity and EV biogenesis using *Synechococcus elongatus* PCC 7942 as a model. By overexpressing RpaA, we aim to concurrently enhance bioluminescence signals and rhythmic EV production, thereby validating RpaA as a key driver of both events. Ultimately, this work seeks to establish a mechanistic link between the circadian oscillator and the secretory machinery, providing a theoretical and experimental foundation for using engineered cyanobacteria as sustainable, photosynthetic cell factories for the rhythmic delivery of bioactive compounds.

## 2. Materials and Methods

### 2.1. Strains and Growth Conditions

The wild-type (WT) strain of *Synechococcus elongatus* PCC 7942 was obtained from the Institute of Hydrobiology, CAS (Wuhan, China). The cyanobacterial strains were cultured in BG11 medium with minor modifications [29,30], either as solid cultures on agar plates or as liquid cultures, supplemented with relevant antibiotics as required [18,31]. All cyanobacterial strains were cultivated at a constant temperature of 30 °C and illuminated continuously with cool-white fluorescent lamps at an intensity of 40~50 μE·m^−2^s^−1^ (LL conditions). For circadian synchronization purposes, cultures were subjected to one or two cycles of alternating 12 h illumination and 12 h darkness (LD 12:12) prior to transferring into constant light conditions. The competent TOP10 *Escherichia coli* cells, used for molecular cloning and plasmid construction, were purchased from Weidi Biotechnology (Shanghai, China). *Escherichia coli* (*E. coli*) transformants were propagated in Luria–Bertani (LB) broth or on LB agar plates [32,33] at 37 °C, with appropriate antibiotics added.

### 2.2. Construction of Bioluminescence Reporter Strains and Mutants

*P_psbAI_*::*luxCDABE* and *P_kaiBC_*::*luxCDABE* bioluminescence reporter cassettes coupled with a Kanamycin-resistance gene for selection were integrated into the neutral site II (NSII) locus on the chromosome of WT *Synechococcus elongatus* PCC 7942 through homologous recombination to generate the base reporter strain 7942 WT [34,35]. The plasmid pGEN-*luxCDABE*, which served as the source of the *luxCDABE* operon, was a generous gift from Harry Mobley (Addgene plasmid # 44918; http://n2t.net/addgene:44918 accessed on 20 September 2024; RRID:Addgene_44918; Michigan, USA) [36]. The *rpaA* deletion mutant (*rpaA*-KO) was subsequently constructed by replacing the entire *rpaA* open reading frame (ORF) with a Spectinomycin-resistance cassette in the 7942 WT background. To generate the IPTG-inducible *rpaA*-OX strain, the native *rpaA* gene was first disrupted by replacing it with a Spectinomycin-resistance cassette. Subsequently, a rescue construct comprising the *rpaA* gene under the control of an IPTG-inducible *Ptrc* promoter and a Gentamicin-resistance marker was integrated into neutral site III (NSIII) [26] (Table S1). All cyanobacterial transformations were performed using the natural transformation method, and transformants were selected on BG11 agar plates containing the appropriate antibiotics using the top agar technique [37]. To ensure complete chromosomal segregation, transformants were re-streaked onto selective agar plates for four to five passages. Full segregation of mutants was subsequently verified by PCR using gene-specific primers flanking the modified loci. All DNA sequences used for constructing mutant strains in this study are provided in Text S1.

### 2.3. Bioluminescence Monitoring

The 7942 WT and *rpaA* mutant strains harboring bioluminescence reporters were cultivated on solid BG11 medium containing the necessary modifications and supplemented with selective antibiotics. Cultures were generally maintained at 30 °C under constant cool-white fluorescent light (LL) at an intensity of 40~50 μE·m^−2^s^−1^. For experimental preparation, actively growing colonies maintained under LL for two to three days were transferred using sterile toothpicks onto freshly prepared agar plates. Following an additional one to two days of growth under illumination, cultures were exposed to a single 12 h dark period to entrain and synchronize the circadian clocks for all the cells. Subsequently, the free-running period (FRP) of *lux* bioluminescence, defined as the cycle length of the rhythmic bioluminescence signal from cyanobacterial colonies, was determined under constant light (LL) conditions without external temporal cues. The measurement was using an automated plant photon-counting instrumentation (3D fluorescence imaging system NightShade LB985; Berthold Technologies, Bad Wildbad, Germany) [34,38,39], which integrates a TopCount-style photon detection camera with a computer-controlled data acquisition system similar to KondoTron [38] (Figure S1).

### 2.4. Extracellular Vesicle Sampling and Quantification

Seed culture of each strain was inoculated at 2% (*v*/*v*) into 150 mL BG11 medium and grown at 30 °C under continuous shaking (150 rpm) with 40~50 μE·m^−2^s^−1^ cool-white light illumination for 1~2 days until reaching mid-log phase (OD_730_ ≈ 0.5). Cultures were then adapted in darkness for 12 h to synchronize circadian rhythms before being transferred to either continuous light (LL) or 12 h:12 h light–dark cycles (LD). Samples were collected every 6 h over 48 h starting from Circadian Time 12 (CT12) or Zeitgeber Time 12 (ZT12), respectively. At each timepoint, 10 mL culture aliquots were centrifuged (6500× *g*, 15 min, 4 °C), and supernatants were filtered through 0.22 µm filter membranes to remove cells and debris. Filtered supernatants were ultracentrifuged (267,000× *g*, 1 h, 4 °C), and pellets were resuspended in EV buffer (Table S6).

For transmission electron microscopy (TEM), a 5 µL purified EV suspension was applied to carbon-coated copper grids, air-dried for 2 min, negatively stained with 2% phosphotungstic acid (pH 6.8) for 2 min, and imaged using a JEOL JEM-2100F TEM (Hitachi, Ltd., Tokyo, Japan) at 80 kV accelerating voltage (Figure S2).

EVs were labeled with lipophilic dye PKH67 by mixing 100 µL of EV suspension with an equal volume of PKH67 working solution (2 µM final concentration in PBS), which was incubated at 37 °C for 5 min, followed by 4 °C for 15 min. Staining was quenched with 1% BSA, and samples were centrifuged (10,000× *g*, 30 min, 4 °C), washed 1~2 times with PBS, and resuspended. Stained EVs were diluted to ~10^7^ particles mL^−1^ and imaged using an Axio Imager Z2 fluorescence microscope (Carl Zeiss AG, Oberkochen, Germany) with EGFP channel excitation 488 nm and emission 509 nm at 40× magnification (Figure S3) [40,41]. Three random fields per slide were analyzed using ImageJ software (IJ 1.46r). EV concentration was calculated as: (average count per field·dilution factor)/(field volume·OD_730_), with yield expressed as particles·OD^−1^_730_·mL^−1^. Data represent the mean ± SD of five biological replicates with three technical replicates each.

### 2.5. Total RNA Extraction and Quantitative Real-Time PCR (QPCR)

Total RNA extracted from each sample was reverse transcribed into complementary DNA (cDNA) using a commercial reverse transcription kit (HiScript III RT SuperMix for QPCR, Vazyme, Nanjing, China), according to the manufacturer’s instructions. Quantitative real-time PCR (QPCR) was subsequently performed to assess the transcript abundance of targeted genes, including *rpaA*, *glgC*, and *lpxD*. Gene-specific primers for each target were designed using Primer3 software (Release 2.6.0) and validated for amplification efficiency and specificity prior to the experiment. The 16S rRNA gene was employed as the internal reference gene for normalization purposes due to its stable expression across experimental conditions (primer sequences are listed in Table S5). Relative gene expression levels were calculated using the comparative Ct method (2^−ΔΔCt^) as previously described [42]. For each target gene, the ΔCt value was first determined by subtracting the Ct value of the 16S rRNA reference gene from that of the target gene. Subsequently, the ΔΔCt was calculated by normalizing all samples to the WT strain harvested at Circadian Time 12 (CT12), whose relative expression level was set to 1. This normalization strategy enabled direct comparison of gene expression changes across different mutant strains and time points relative to the WT reference condition. All QPCR reactions were performed with five biological replicates, and data are presented as mean ± standard deviation from these five independent biological replicates accordingly.

### 2.6. Period and Phase Analyses with the Lux Reporter Strains and the QPCR Data

Circadian period lengths of bioluminescence rhythms and rhythmic gene expression patterns were determined using the BioDare2 online platform https://biodare2.ed.ac.uk/ (accessed on 6 November 2025), a publicly accessible web-based tool for the analysis of biological time-series data [43]. Raw bioluminescence traces obtained from the Topcount detection system, the EV secretion patterns, and QPCR-derived relative expression values collected at multiple time points were uploaded to the BioDare2 server in the appropriate format based on the instructions. Period estimation and phase calculation (peak of the event) were performed using the mFourfit algorithm, which provides robust detection of circadian periodicities even with noise or damping. All periodic values are presented in the form of mean ± standard deviation, with each group of data obtained from at least 3 independent biological replicates.

## 3. Results

### 3.1. Experimental Design

The study involved multiple *Synechococcus elongatus* PCC 7942 strains: the wild-type (WT), an *rpaA*-null mutant (*rpaA*-KO), and an *rpaA* overexpression strain (*rpaA*-OX) induced with isopropyl β-D-1-thiogalactopyranoside (IPTG) at several concentrations (0 mM, 0.1 mM, 0.5 mM, 1 mM, 5 mM and 10 mM). Only the cultures induced with 1 mM IPTG were utilized for EV isolation and calculation, as well as for the subsequent QPCR assays.

**Figure 1:**
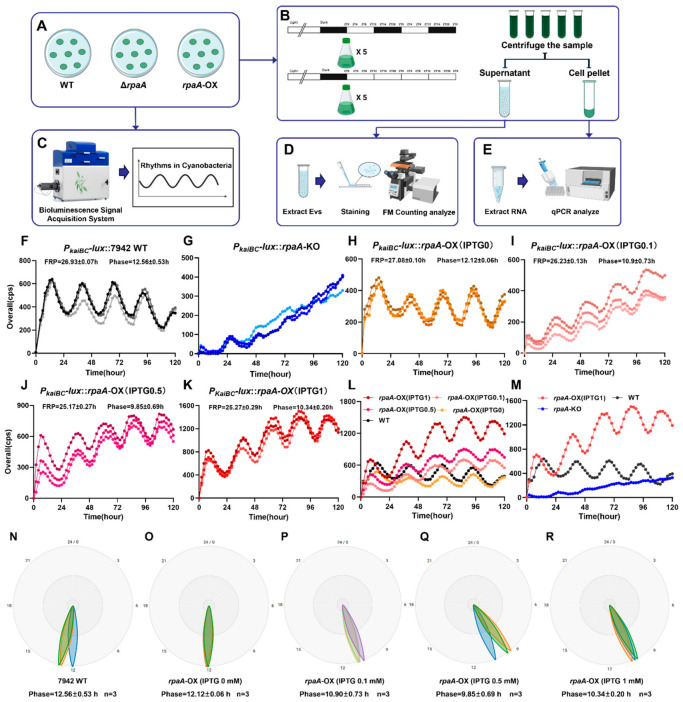
Overview of experimental design and bioluminescence rhythmicity in wild-type and *rpaA* mutant strains. (**A**) The *rpaA*-knockout (*rpaA*-KO) and -overexpression (*rpaA*-OX) strains were constructed in the background of the wild-type strain (WT), which has integrated the *luxCDABE* reporter system. (**B**) Each strain was cultivated in shake flasks under LD and LL, with sampling continuously every 6 h over 48 h. (**C**) All strains were cultured at 30 °C under LL (40~50 µE/m^2^s), and bioluminescence rhythms were monitored for 120 h using the Topcount detection system. (**D**) EVs in the supernatant were extracted, stained and quantified via fluorescence microscopy counting. (**E**) RNA was extracted from algal pellets, and transcriptional analysis was performed by QPCR. (**F**) Bioluminescence rhythms of WT in the *P_kaiBC_*::*luxCDABE* background under LL (black). (**G**) Complete loss of rhythmicity in the *rpaA*-KO (blue). (**H**–**K**) Dose-dependent bioluminescence rhythms in the *rpaA*-OX under 0 (orange), 0.1 (pink), 0.5 (purple), and 1 mM (red) IPTG concentrations. Three representative traces are shown with free-running period (FRP) values indicated in each panel. (**L**) Overlay comparison of bioluminescence rhythms from *rpaA*-OX induced with 0 mM (orange), 0.1 mM (pink), 0.5 mM (purple), and 1 mM IPTG (red), alongside WT (black). (**M**) Comparison of bioluminescence rhythms among *rpaA*-OX (1 mM IPTG, red), *rpaA*-KO (blue), and WT (black) under LL. Data are presented as mean ± SD (*n* = 3); statistical analysis was performed using ordinary one-way (ANOVA) in GraphPad Prism (version 8.2.1), *p* < 0.05. (**N**–**R**) Phase analysis of WT and *rpaA*-OX strains in the *P_kaiBC_*::*luxCDABE* background. The raw bioluminescence traces data were uploaded to the BioDare2 server with a time range of 0–120 h and data category set to Luc/GFP-imaging. After linear detrending (DTR), the mFourfit algorithm was applied for phase analysis (*n* = 3).

To capture stabilized rhythmic signals, we utilized Circadian Time (CT) and Zeitgeber Time (ZT) to track internal and environmental timing, respectively. The cells were cultivated under two environmental regimes: LL and LD. Sampling was performed at 6 h intervals over a 48 h period, beginning at Circadian Time 12 (CT12) for cultures maintained in LL or at Zeitgeber Time 12 (ZT12) for those grown under LD, which was 12 h after two 12 h:12 h LD entrainment cycles (Figure 1A–C). This 12 h buffer period before sampling was implemented to bypass transient signal instability often observed immediately after phase resetting. Continuous monitoring for 48 h thereafter ensured the capture of two full free-running cycles. At each sampling time point, liquid cultures were centrifuged to separate the liquid phase, which was subsequently used to quantify EVs (Figure 1D). The corresponding cell pellets were collected for total RNA extraction and subsequent quantitative PCR (QPCR) analysis (Figure 1E). For each time point and each experimental condition, five independent biological replicates were included to ensure statistical robustness and reproducibility.

### 3.2. Regulatory Contribution of Global Transcription Factor RpaA to Rhythmicity

To monitor circadian rhythmicity, two *luxCDABE*-based bioluminescent reporter strains driven by distinct promoters were constructed. The reporter cassettes *P_psbAI_*::*luxCDABE* and *P_kaiBC_*::*luxCDABE* were individually integrated into the neutral site II (NSII) locus on the chromosome of the WT *Synechococcus elongatus* PCC 7942, thereby generating the base strain 7942 WT [34,35]. Both *P_psbAI_*::*lux* and *P_kaiBC_*::*lux* are standard, robust reporters for monitoring 7942 rhythms. To ensure that our findings were not promoter-specific, we employed both systems for cross-verification. Results obtained with *P_kaiBC_*::*lux* are featured in the main text, while *P_psbAI_* data are provided in the Supplementary Materials. Both the *rpaA*-deletion mutant (*rpaA*-KO) and the IPTG-inducible *rpaA* overexpression strain (*rpaA*-OX) were subsequently constructed in this 7942 WT background (Tables S2–S4), allowing for the direct comparison of bioluminescence output. Here, we present the bioluminescence rhythms of wild-type and mutant strains in the *P_kaiBC_*::*luxCDABE* background. In addition, we also show the bioluminescence rhythms and phase analysis of each strain in the *P_psbAI_*::*luxCDABE* background in the Supplementary Materials (Figures S4 and S5), where both reporter systems produced comparable results.

Under LL conditions, the 7942 WT strain displayed robust circadian oscillations with a period of approximately 24 h, which was consistent with previous characterizations of the cyanobacterial circadian clock [9,34] (Figure 1F). As anticipated from prior studies demonstrating the essential role of RpaA as a master output regulator of the Kai oscillator [9,10], the *rpaA*-KO mutant exhibited completely arrhythmic bioluminescence patterns, confirming that RpaA is indispensable for transmitting temporal information from the core oscillator to downstream gene expression (Figure 1G).

Notably, the IPTG-inducible *rpaA*-OX strain revealed a clear dosage-dependent effect on circadian output. When IPTG concentrations were increased incrementally from 0 mM, 0.1 mM, 0.5 mM, and then to 1 mM, the amplitude of *lux* bioluminescence exhibited a corresponding gradual elevation. This positive correlation between inducer concentration and bioluminescence intensity strongly suggested that the cellular abundance of RpaA was directly and positively associated with the amplitude of circadian output signal transmission (Figure 1H–L). Compared with the 7942 WT strain, *rpaA*-OX not only maintained a robust 24 h bioluminescent rhythm but also exhibited a higher bioluminescent amplitude. In contrast, *rpaA*-KO had a significantly reduced luminescence level, which was also arrhythmic (Figure 1M). These findings indicate that RpaA protein levels might serve as a rate-limiting factor in determining the strength of clock-controlled gene expression. Particularly, the *rpaA*-OX strain displayed rhythmic oscillations even under non-inducing conditions (0 mM IPTG). This phenotype stems from promoter leakiness, as confirmed by QPCR data demonstrating that basal *rpaA* transcript levels in the IPTG-untreated group were comparable to those in the wild type (WT) (Figure S6).

To analyze circadian phases, raw bioluminescence trajectory data collected from the automated fluorescence detection system were imported into BioDare2. The circadian phase of the WT was approximately 12.56 h. For the *rpaA*-OX strain, the phases were approximately 12.12 h (0 mM IPTG), 10.9 h (0.1 mM IPTG), 9.85 h (0.5 mM IPTG), and 10.34 h (1.0 mM IPTG). Overall, both the WT and *rpaA*-OX strains maintained a clustered peak phase between CT 10 and CT 12, indicating that modulating *rpaA* expression levels does not cause a drastic phase shift in the circadian clock (Figure 1N–R).

### 3.3. Association Between RpaA Abundance and Bioluminescence Amplitude

To investigate the relationship between RpaA protein levels and bioluminescence output, we strategically modulated RpaA expression across a broad range of inducer concentrations. Based on preliminary observations, we hypothesized that RpaA enhances bioluminescence at physiological or moderate concentrations but exerts an inhibitory effect at hyper-physiological levels.

**Figure 2:**
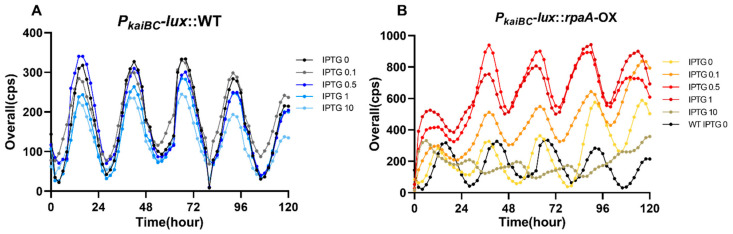
RpaA abundance and bioluminescence amplitude correlations. (**A**) Comparison of the relationship between IPTG induction and bioluminescence amplitude in the WT strain. IPTG treatment exerted no discernible effect on the bioluminescence amplitude of the WT strain across all tested concentrations. (**B**) Correlation between IPTG-induced RpaA expression level and bioluminescence amplitude. For the *rpaA*-OX strain, the amplitude increased in a dose-dependent manner with IPTG concentrations below 1 mM, reflecting a correlation with enhanced RpaA abundance. Conversely, once the IPTG concentration reached a high dose of 10 mM, the amplitude decreased drastically. A representative trace is shown for each IPTG concentration.

To test this hypothesis, the *rpaA*-OX strain was induced with an IPTG gradient (0, 0.1, 0.5, 1, and 10 mM), while the wild-type (WT) strain subjected to the same regime served as a control. Crucially, IPTG treatment exerted no discernible effect on the bioluminescence amplitude of the WT strain across all tested concentrations (Figure 2A), ruling out any confounding physiological stress induced by the chemical itself. In contrast, for the *rpaA*-OX strain, the amplitude increased in a dose-dependent manner with IPTG concentrations below 1 mM, reflecting a correlation with enhanced RpaA abundance. Conversely, once the IPTG concentration reached a high dose of 10 mM, the amplitude decreased drastically, successfully validating our biphasic dosage hypothesis (Figure 2B).

These results indicate that an optimal, elevated level of RpaA promotes bioluminescence output, whereas extreme overexpression disrupts rhythmicity and reduces amplitude.

### 3.4. Rhythmic Secretion of Extracellular Vesicles in WT and rpaA Mutants

**Figure 3:**
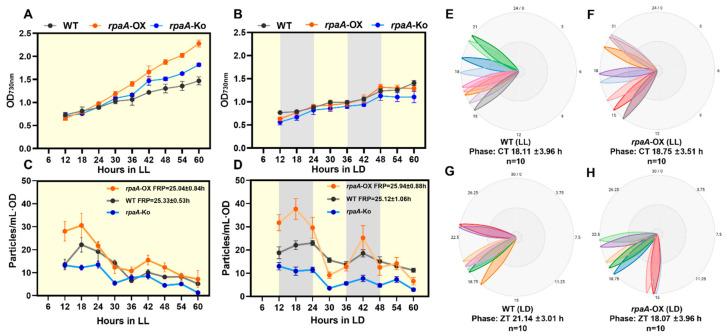
RpaA modulated rhythmic EV secretion. (**A**,**B**) Growth curves under LL and LD conditions over 48 h, monitored by optical density at 730 nm (OD_730_), the yellow area in the panel represents LL conditions, and the alternating yellow and gray parts indicate LD conditions. (**C**,**D**) Rhythmic EV secretion profiles under LL and LD conditions over 48 h. EV yield was normalized and is expressed as particles·OD^−1^_730_·mL^−1^. (**E**–**H**) The raw data of 48 h EV secretion in WT and *rpaA*-OX strains under LL and LD conditions were imported into the BioDare2 server. The time range was set to 0–48 h, and the data category was set to biomass. After DTR treatment, the mFourfit algorithm was selected for phase analysis. Data represent mean ± SD (*n* = 10) from five biological replicates with two technical replicates each, *p* < 0.05.

To test whether extracellular vesicle (EV) yield follows a similar trend to bioluminescence in response to increasing RpaA abundance, we selected the wild-type (WT), *rpaA*-KO, and *rpaA*-OX strains for EV quantification. The *rpaA*-OX strain was induced with 1 mM IPTG, a concentration at which bioluminescence was previously observed to be strongest. The cells cultivated under LL conditions exhibited faster growth rates compared to those under LD conditions in general. Notably, the *rpaA*-OX strain demonstrated the most rapid growth under LL conditions, whereas the *rpaA*-KO mutant displayed the slowest growth rate under LD conditions, a phenotype consistent with previously published observations regarding the essential role of RpaA in regulating cellular fitness under cyclic environments [9,22] (Figure 3A,B).

The EV secretion dynamics revealed pronounced differences among the strains tested. Both the WT and *rpaA*-OX strains exhibited robust and rhythmic EV production under both LD and LL conditions. Furthermore, the *rpaA*-OX strain consistently produced substantially higher quantities of EVs compared to WT across all time points examined. In contrast, the *rpaA*-KO mutant not only failed to display any detectable rhythmicity in EV secretion under either LD or LL, but also exhibited dramatically reduced overall EV production, suggesting RpaA’s critical role in both the temporal regulation and amplitude of EV biogenesis (Figure 3C,D).

Phase analysis of EV secretion showed that the WT reached its secretion peak at Circadian Time 18 (CT18) under LL conditions, and at Zeitgeber Time 21 (ZT21) under LD conditions. By contrast, the *rpaA*-OX strain exhibited secretion peaks at CT18 and ZT18 (Figure 3E–H). These findings are consistent with previous reports that EV secretion displays an elevated pattern during the nighttime [20]. Since the *rpaA*-KO strain completely lost the rhythmicity of EV secretion, no phase analysis data were obtained for this strain. Importantly, while the *rpaA*-OX strain showed markedly elevated amplitude of EV secretion, the phase of the secretion rhythm remained unchanged relative to the WT. These findings suggest that elevated RpaA abundance enhances the output strength of clock-controlled EV secretion without altering its temporal coordination with the underlying circadian oscillator.

### 3.5. Transcripts of Genes Regulating Extracellular Vesicles and Carbohydrate Production

Quantitative real-time PCR (QPCR) was employed to evaluate the relative transcript abundances of selected genes across the WT, *rpaA*-KO, and *rpaA*-OX strains, using the 16S rRNA gene as the internal reference for normalization. The target genes examined included *rpaA* itself, *glgC* (encoding ADP-glucose pyrophosphorylase, a key enzyme in glycogen biosynthesis) [22], and *lpxD* (encoding UDP-3-O-acyl-glucosamine N-acyltransferase, which is involved in lipopolysaccharide biosynthesis and implicated in extracellular vesicle biogenesis through facilitating membrane blebbing and EV release) [44,45].

**Figure 4:**
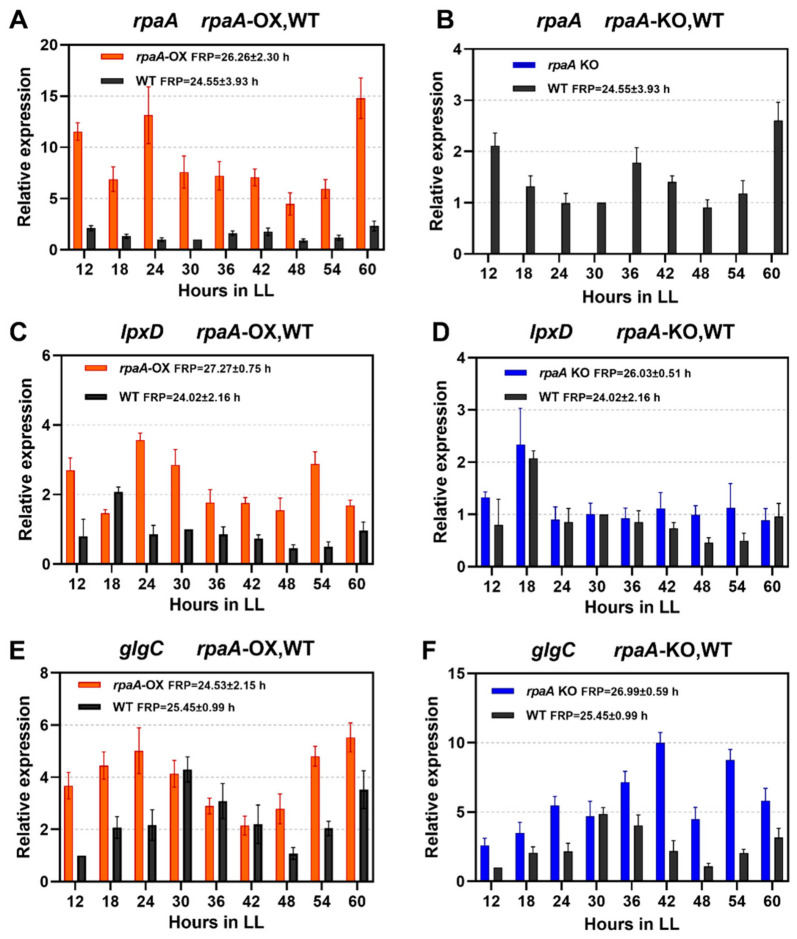
QPCR analysis of target gene transcription in *rpaA*-KO, *rpaA*-OX and WT strains. (**A**,**B**) Transcript abundance of *rpaA* in *rpaA*-OX (orange), WT (black) and *rpaA*-KO (blue) strains. (**C**,**D**) Transcript abundance of *lpxD* in each strain. (**E**,**F**) Transcript abundance of *glgC* in each strain. Free-running period (FRP) values for gene expression rhythms are indicated in each panel. Data represent mean ± SD (*n* = 5), *p* < 0.05.

As expected, *rpaA* transcript levels were approximately 4.99-fold higher in the *rpaA*-OX strain compared to WT, confirming successful overexpression of *rpaA* (Figure 4A). Conversely, no detectable *rpaA* mRNA was observed in the *rpaA*-KO mutant, validating complete deletion (Figure 4B). With respect to *lpxD* expression, no significant alterations were detected in the *rpaA*-KO background relative to WT (Figure 4D); however, a notable increase in *lpxD* transcript abundance was observed in the *rpaA*-OX strain (Figure 4C), suggesting a potential positive regulatory relationship between RpaA and genes involved in membrane or EV biogenesis, which was also consistent with the increased secretion of EVs in *rpaA*-OX.

Intriguingly, *glgC* expression exhibited a distinct pattern: while the *rpaA*-OX strain displayed only a modest increase in *glgC* mRNA levels (Figure 4E), the *rpaA*-KO mutant showed a substantially elevated accumulation of *glgC* transcripts compared to WT (Figure 4F). This observation is consistent with previous reports indicating that disruption of circadian output signaling leads to metabolic dysregulation and enhanced glycogen accumulation in cyanobacteria [22,46]. These results suggest that, although the loss of RpaA abolishes circadian rhythmicity in clock-controlled gene expression, the capacity for glycogen biosynthesis and storage remains intact or may even be enhanced under arrhythmic conditions.

## 4. Discussion

This study provides novel insights into the role of the master circadian output regulator RpaA in modulating both bioluminescence rhythmicity and extracellular vesicle (EV) biogenesis in *Synechococcus elongatus* PCC 7942. Our findings not only validate previous observations regarding RpaA function but also extend our understanding of how circadian clock manipulation can be leveraged for biotechnological applications.

Consistent with earlier reports [9,10,15], deletion of *rpaA* resulted in complete loss of circadian bioluminescence rhythmicity, confirming the indispensable role of RpaA in transducing clock information from the KaiABC oscillator to downstream transcriptional outputs. However, overexpression of *rpaA* did not disrupt circadian rhythmicity. Instead, the *rpaA*-OX strain maintained robust oscillations while exhibiting substantially enhanced bioluminescence amplitude in an IPTG dose-dependent manner. Notably, this relationship was biphasic: bioluminescence amplitude increased with IPTG concentrations up to 1 mM but declined sharply when IPTG exceeded 2 mM, indicating that an optimal RpaA level is required for maximal output. These observations indicate that RpaA abundance functions as a rate-limiting factor that determines the amplitude of the oscillation. The preservation of rhythmicity in the *rpaA*-OX strains further suggests that elevated RpaA levels within an optimal concentration range do not saturate the circadian signaling pathway, but instead amplify the output signal proportionally. Beyond a certain window, the system becomes overburdened and then arrhythmic. The strategic role of RpaA observed in our study aligns with recent findings from Golden and colleagues, who showed that increasing phosphorylated RpaA concentrations promotes *PkaiBC* transcription in vitro, providing a mechanistic parallel to our in vivo observations [47].

Extending these findings to EV biology, we demonstrated that both WT and *rpaA*-OX strains exhibited robust rhythmic EV secretion under both LL and LD conditions, with peak production occurring at ZT18 and CT18, respectively. This temporal pattern aligns with recent reports documenting circadian regulation of EV release in cyanobacteria [20]. Importantly, *rpaA*-OX strains maintained identical phase characteristics to WT while producing significantly higher quantities of EVs, reinforcing the conclusion that RpaA amplifies output amplitude without altering circadian phase. Conversely, the *rpaA*-KO mutant exhibited both arrhythmic and dramatically reduced EV production, establishing RpaA as a critical determinant of both the timing and quantity of EV biogenesis. While chromosomal copy numbers in *Synechococcus elongatus* can vary based on growth phase, ranging from 4–10 copies in lag phase to fewer in exponential growth, they lack the phase-locked periodicity observed here [48]. In our *rpaA*-OX strain, bioluminescence and EV yields maintain stable, growth-independent peaks at CT12 and CT18, respectively. This circadian precision across varying stages confirms that the rhythmic enhancement is driven by specific RpaA-mediated transcriptional activation rather than stochastic fluctuations in chromosomal copy number.

At the transcriptional level, elevated *lpxD* expression in *rpaA*-OX strains provides a potential mechanistic explanation for enhanced EV production, as LpxD participates in lipopolysaccharide biosynthesis, a pathway intimately linked to outer membrane EV formation in Gram-negative bacteria [45]. The positive correlation between RpaA abundance, *lpxD* transcription, and EV secretion suggests that RpaA may directly or indirectly activate genes involved in membrane lipid metabolism. Intriguingly, *glgC* expression exhibited an opposite pattern, with substantially elevated transcript levels in the *rpaA*-KO mutant. This finding is consistent with previous observations that arrhythmic cyanobacterial strains accumulate excess glycogen due to metabolic dysregulation [22,46]. The divergent responses of *lpxD* and *glgC* to RpaA perturbation suggest that lipid and carbohydrate metabolism may be subject to distinct regulatory mechanisms within the circadian output network, with RpaA preferentially promoting lipid-related pathways while its absence derepresses carbohydrate storage.

Previous studies have demonstrated that manipulation of circadian clock components, such as the overexpression of *kaiA* or the deletion of *labA*, can substantially increase heterologous gene expression and metabolite production [25,26]. Our results establish *rpaA* overexpression as an additional and complementary approach that not only enhances the production of functionally versatile EVs but may also augment the yield of other clock-controlled gene products.

## 5. Conclusions

This study identifies RpaA as the master regulator coordinating both bioluminescence and EV biogenesis in *Synechococcus elongatus* PCC 7942. It is demonstrated that RpaA abundance is a rate-limiting factor that dictates the strength of circadian outputs. Its overexpression significantly boosts EV yield by optimizing carbon allocation and membrane-related transcription. These findings establish circadian engineering as a powerful strategy for enhancing the productivity of cyanobacterial cell factories, providing a robust framework for the sustainable production of engineered nanocarriers in biotechnological and therapeutic applications.

## Data Availability

The datasets generated and/or analyzed during the present study are provided in this article and the Supplementary Materials. Additional data are available from the corresponding author upon reasonable request.

## Supplementary Materials

The following supporting information can be downloaded at: https://www.mdpi.com/article/10.3390/life16060885/s1, Figure S1: Monitoring the bioluminescence rhythm of reporter strains using a TopCount-style photon detection camera. (**A**) The model of bioluminescence signal acquisition and rhythm monitoring. (**B**–**D**) The wild-type reporter strain 7942-WT’s luminescence detected at CT4, CT8 and CT12 points under a exposure time of 250 s; Figure S2: Morphological identification of EVs by TEM (JEOL JEM-2100F). The accelerating voltage of the transmission electron microscope (TEM) was adjusted to 80 Kv, use high-power lens (×60 k~120 k) to observe clear images, The diameter of the observed extracellular vesicles is approximately 80~200 nm; Figure S3: Morphological identification of EVs by FM (Axio imager Z2). The upright fluorescence microscope was switched to the EGFP channel, the excitation wavelength was 488 nm and emission wavelength was 509 nm. The objective lens focal length was adjusted (40× selected and 100× selected). All images were processed to remove background noise. (**A**–**D**) Observation of S.elongatus PCC 7942,The results were observed under the EGFP channel and Bright channel after magnification by 40× and 100× respectively, with the red arrows indicating the stained cyanobacteria. (**E**–**H**) Observation of EVs, with the yellow arrows indicating the stained EVs; Figure S4: Overview of bioluminescence rhythmicity in wild-type and *rpaA* mutant strains of *P_psbAI_*::*luxCDABE* background. (**A**) Bioluminescence rhythms of wild-type (WT) *Synechococcus elongatus* PCC 7942 colonies under LL. (**B**) Complete loss of rhythmicity in the *rpaA*-KO mutant strain. (**C**–**F**) Dose-dependent bioluminescence rhythms in the *rpaA*-OX strain under different IPTG concentrations (0, 0.1, 0.5, and 1 mM). Three representative traces are shown with FRP values indicated in each panel. (**G**) Overlay comparison of bioluminescence rhythms from *rpaA*-OX strains induced with 0 mM (pale yellow), 0.1 mM (deep yellow), 0.5 mM (orange), and 1 mM IPTG (red), alongside WT (black). Bioluminescence amplitude showed a positive correlation with IPTG concentration. (**H**) Comparison of bioluminescence rhythms among *rpaA*-OX (1 mM IPTG, orange), *rpaA*-KO (blue), and WT (black) strains under LL conditions. Data are presented as mean ± SD (n = 3), statistical analysis was performed using Ordinary one-way (ANOVA) in GraphPad Prism, p < 0.05; Figure S5: Phase analysis of WT and *rpaA*-OX strains in the *P_psbAI_*::*luxCDABE* background. (**A**) Bioluminescence phase of the WT strain. (**B**–**E**) Bioluminescence phase of the *rpaA*-OX strain under different IPTG concentrations (0, 0.1, 0.5, and 1 mM). Luminescence phases were all around circadian time (CT) 10~12 hours (n = 3); Figure S6: QPCR verification of *rpaA* leaky expression in the *rpaA*-OX strain compared with the WT. For the *rpaA*-OX strain, two experimental groups were evaluated: 1 mM IPTG induction (red bars) and an uninduced control (0 mM IPTG; yellow bars), with the WT strain serving as a baseline control (yellow bars). Continuous sampling was performed over a 48 h period, followed by transcriptional analysis to determine whether *rpaA* leaky expression occurs in the absence of inducer and whether its baseline expression mirrors WT levels. Data are presented as mean ± SD (n = 3); Table S1: *Synechococcus elongatus* PCC 7942 strains used in this study; Table S2: Primers for *luxCDABE* reporter plasmid construction; Table S3: Primers for *rpaA* knockout plasmid construction**;** Table S4: Primers for *rpaA* overexpression plasmid construction; Table S5: Primers for QPCR; Table S6: Extracellular Vesicles Buffer system; Text S1: DNA sequences used for constructing mutant strains.
