# Supplementary material for: RpaA Overexpression Enhances Bioluminescence Intensity and Elevates Rhythmic Extracellular Vesicle Yield in Synechococcus elongatus PCC 7942
Source: Life (Basel). 2026 May 25;16(6):885. doi: 10.3390/life16060885 (PMC13301164; doi:10.3390/life16060885)
Supplement: Supplementary file 1 [file life-16-00885-s001.zip › life-4307962-supplementary.pdf]

Supplementary Materials

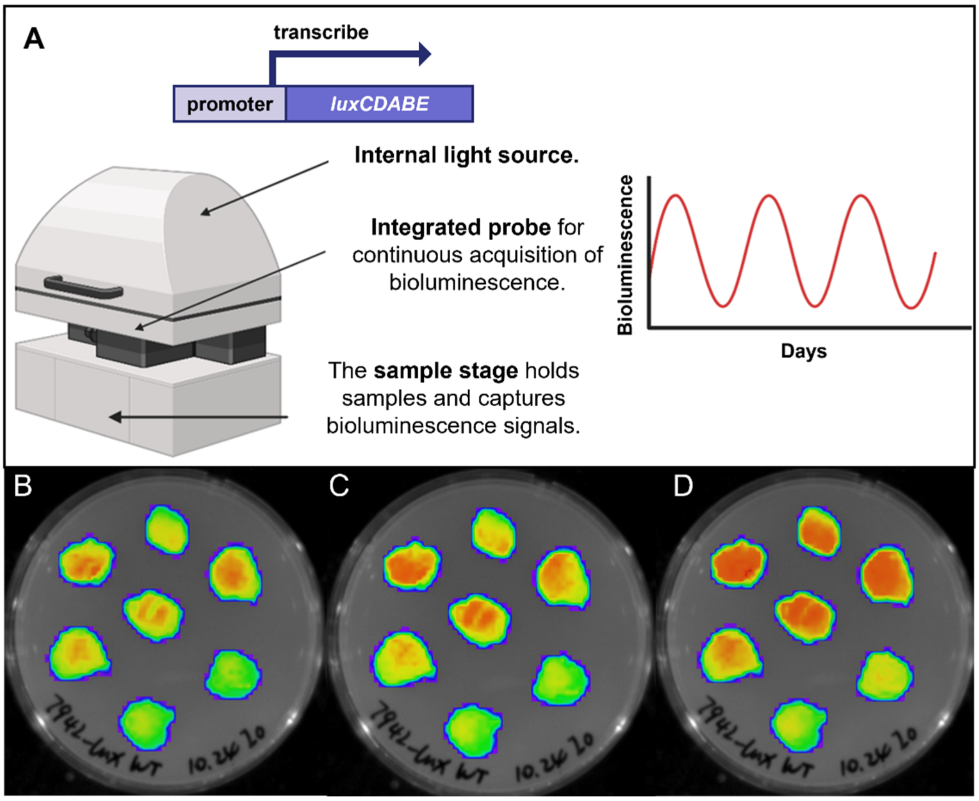

Figure S1. Monitoring the bioluminescence rhythm of reporter strains using a TopCount-style photon detection camera. **(A)** The model of bioluminescence signal acquisition and rhythm monitoring. **(B-D)** The wild-type reporter strain 7942-WT's luminescence detected at CT4, CT8 and CT12 points under a exposure time of 250 s.

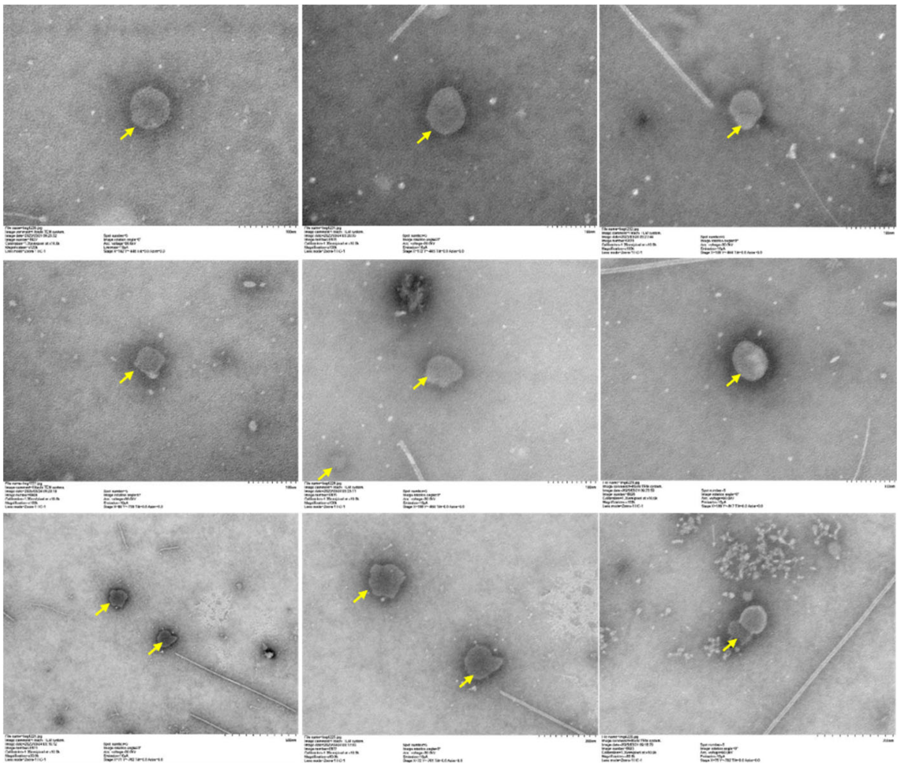

Figure S2. Morphological identification of EVs by TEM(JEOL JEM-2100F). The accelerating voltage of the transmission electron microscope (TEM) was adjusted to 80 Kv, use high-power lens (×60 k~120 k) to observe clear images, The diameter of the observed extracellular vesicles is approximately 80~200 nm.

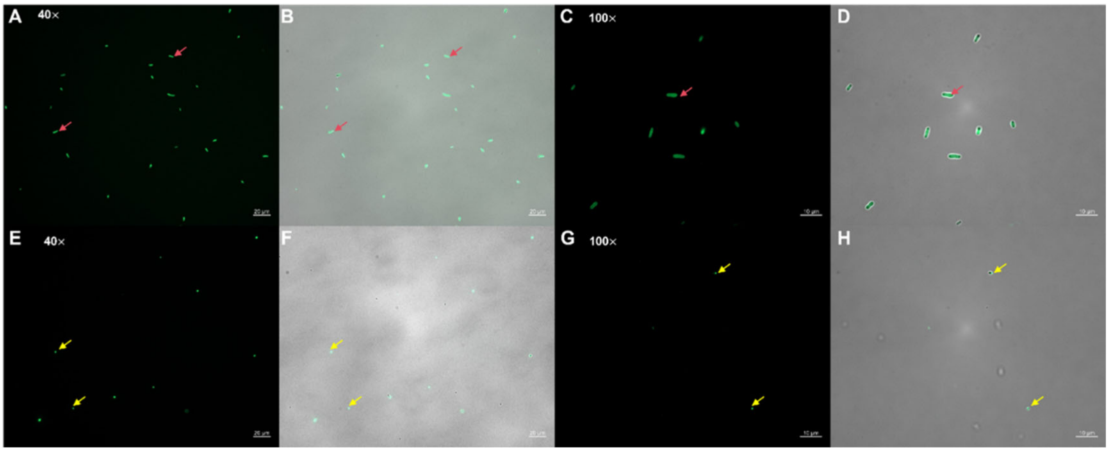

Figure S3. Morphological identification of EVs by FM(Axio imager Z2). The upright fluorescence microscope was switched to the EGFP channel, the excitation wavelength was 488 nm and emission wavelength was 509 nm. The objective lens focal length was adjusted (40× selected and 100× selected). All images were processed to remove background noise. (A-D) Observation of S.elongatus PCC 7942,The results were observed under the EGFP channel and Bright channel after magnification by 40× and 100× respectively, with the red arrows indicating the stained cyanobacteria. (E-H) Observation of EVs, with the yellow arrows indicating the stained EVs.

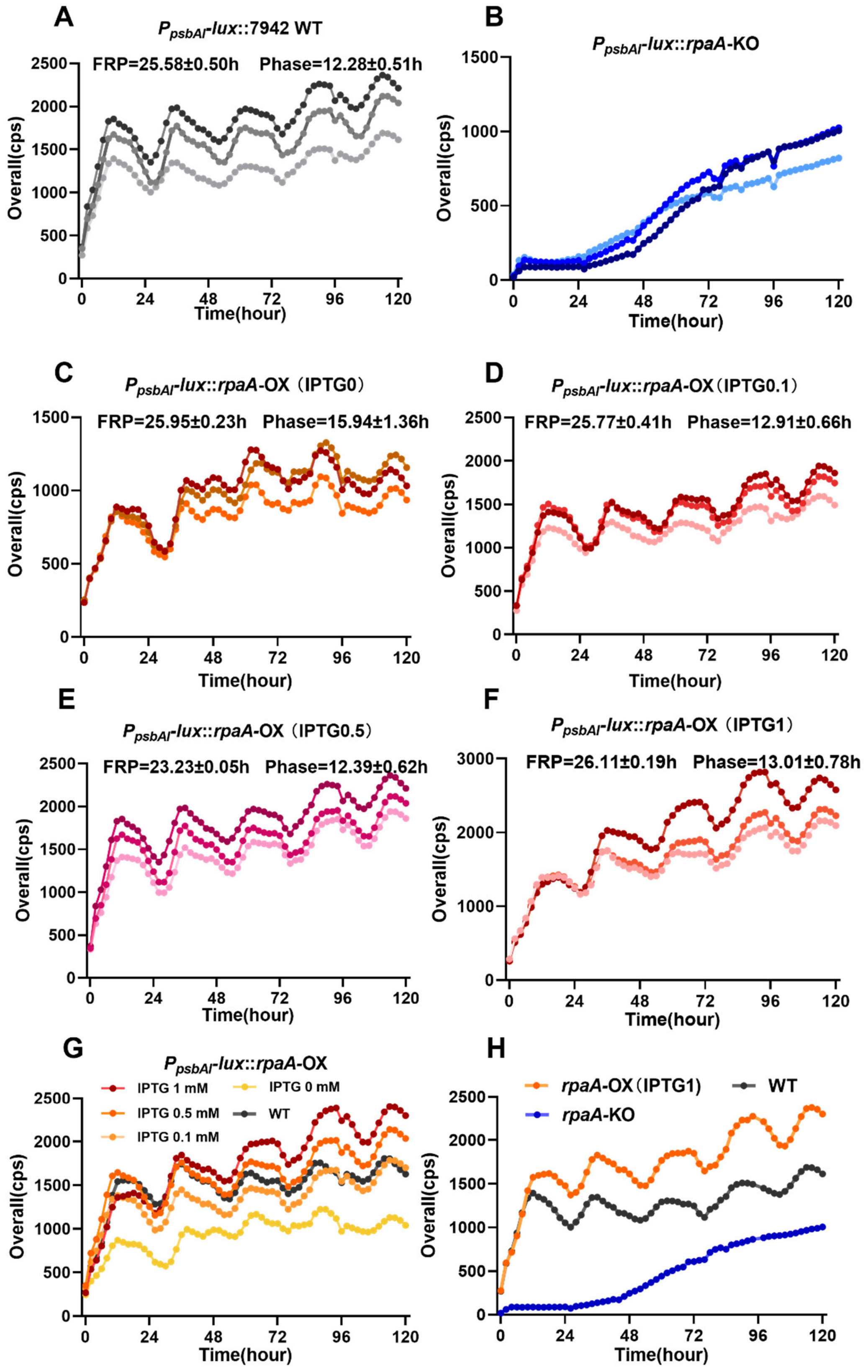

Figure S4. Overview of bioluminescence rhythmicity in wild-type and *rpaA* mutant strains of P_{psbAI}::*luxCDABE* background. **(A)** Bioluminescence rhythms of wild-type (WT) *Synechococcus elongatus* PCC 7942 colonies under LL. **(B)** Complete loss of rhythmicity in the *rpaA*-KO mutant strain. **(C-F)** Dose-dependent bioluminescence rhythms in the *rpaA*-OX strain under different IPTG concentrations (0, 0.1,

0.5, and 1 mM). Three representative traces are shown with FRP values indicated in each panel. **(G)** Overlay comparison of bioluminescence rhythms from *rpaA*-OX strains induced with 0 mM (pale yellow), 0.1 mM (deep yellow), 0.5 mM (orange), and 1 mM IPTG (red), alongside WT (black). Bioluminescence amplitude showed a positive correlation with IPTG concentration. **(H)** Comparison of bioluminescence rhythms among *rpaA*-OX (1 mM IPTG, orange), *rpaA*-KO (blue), and WT (black) strains under LL conditions. Data are presented as mean ± SD (n = 3), statistical analysis was performed using Ordinary one-way (ANOVA) in GraphPad Prism, $p < 0.05$.

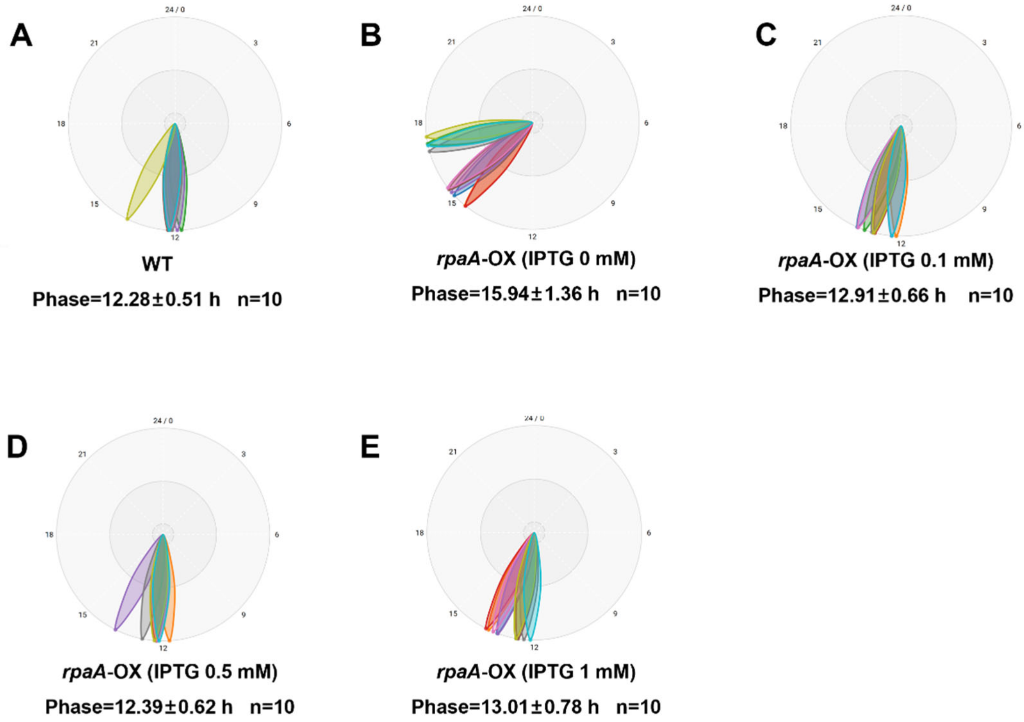

Figure S5. Phase analysis of WT and *rpaA*-OX strains in the P_{psbAI}::*luxCDABE* background. **(A)** Bioluminescence phase of the WT strain. **(B-E)** Bioluminescence phase of the *rpaA*-OX strain under different IPTG concentrations (0, 0.1, 0.5, and 1 mM). Luminescence phases were all around circadian time (CT) 10~12 hours (n = 3).

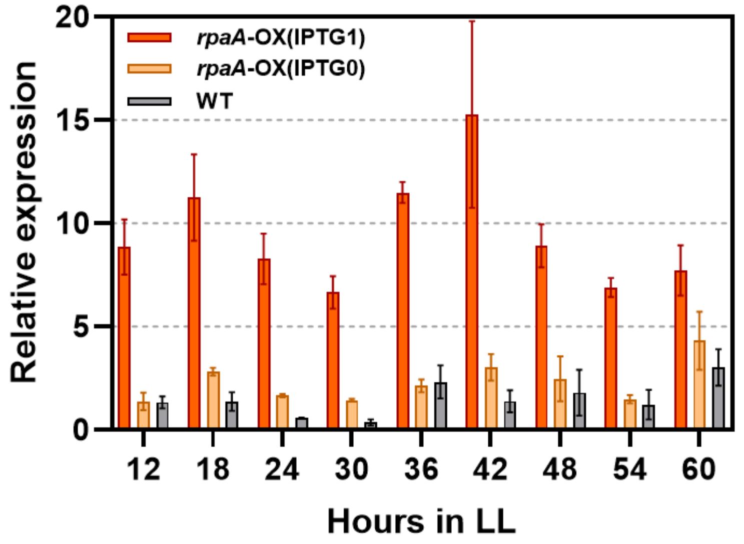

Figure S6. QPCR verification of *rpaA* leaky expression in the *rpaA*-OX strain compared with the WT. For the *rpaA*-OX strain, two experimental groups were evaluated: 1 mM IPTG induction (red bars) and an uninduced control (0 mM IPTG; yellow bars), with the WT strain serving as a baseline control(yellow bars). Continuous sampling was performed over a 48 h period, followed by transcriptional analysis to determine whether *rpaA* leaky expression occurs in the absence of inducer and whether its baseline expression mirrors WT levels. Data are presented as mean ± SD (n = 3).

Table S1. *Synechococcus elongatus* PCC 7942 strains used in this study

Strain	Description	Antibiotic resistance
7942 WT-1	P_{psbAI}::*luxCDABE* at NSII of the wild-type strain	Km^r
7942 WT-2	P_{kaiBC}::*luxCDABE* at NSII of the wild-type strain	Km^r
rpaA-KO-1	P_{psbAI}::*luxCDABE* at NSII of the *rpaA*-null strain	Sp^r; Km^r
rpaA-KO-2	P_{kaiBC}::*luxCDABE* at NSII of the *rpaA*-null strain	Sp^r; Km^r
rpaA-OX-1	P_{trc}-*lacI*::*rpaA* at NSIII and P_{psbAI}::*luxCDABEAB* in NSII of the wild type	Gm^r; Km^r
rpaA-OX-2	P_{trc}-*lacI*::*rpaA* at NSIII and P_{kaiBC}::*luxCDABEAB* in NSII of the wild type	Gm^r; Km^r

Table S2. Primers for *luxCDABE* reporter plasmid construction

Gene name	Forward primer (5' to 3')	Reverse primer (5' to 3')
NSII up	CTCGAGTTTTTCAGCAAGATGA	AGGGATAACTGAGAGTCAACA
NSII down	CCAAATCGTCCGAAAATCAC	CGGATGAGGCAAAACCCTG
Km^r	ATTACACGTCTTGAG	GCGAAGAACTCCAG
P_{psbAI}	ATCCCTGTTTAGTGCGA	TGAGGTTGTAAAGGGC
P_{kaiBC}	TACCCGCCTACGAAAGCC	ACGCAGATCAACGGGG
luxCDABE	GCTTCCATGAAGCTCTTCCA	CGCCGACAACACCATTATCT

Table S3. Primers for *rpaA* knockout plasmid construction

Gene name	Forward primer (5' to 3')	Reverse primer (5' to 3')
rpaA up	ACCCAAGCACTGAGCAGCAC	CAGTCTGCGATCGCCCAACT
rpaA down	TGGGCCTTGCGAGATATGGC	AGGGTAACATTCCGGGAGGA
Sp^r	ATACAGAAGCTGGGCGAA	TCTAGGGTCCCCAATTAATTA

Table S4. Primers for *rpaA* overexpression plasmid construction

Gene name	Forward primer (5' to 3')	Reverse primer (5' to 3')
NSIII up	TCAGCCAGCTCGTCGTG	CGTCCGATCAACCAGTCCCTCATC
NSIII down	CTGACTAGTTCGGTCGACAAGCC	AAGATACAGTCGGCGTCACGGCAA
Gm^r	CGATCTCGGCTTGAACGAAT	TCCTTGTGTATAAGGGGACACTGT
P_{trc}-*lacI*	TCCTTGTGTATAAGGGGACAC	TAATTGTCAATCACTGCCCGC
rpaA	GTGAGCGGATAACAAAGAGGAT	CTAGTCAGGACGCCGAAGGAAACT

Table S5. Primers for QPCR

Gene name	Forward primer (5' to 3')	Reverse primer (5' to 3')
PCC 7942 16S rRNA	AGTTGCCATCATTCAGTTGGGC	CACCTCGCGGCTTCGCGTCT
glgC	CAGATTACGCCGGAGAACCCCA	CTGAATGAACTGGCTATAGTCC
rpaA	GCTGACCGCCCTCGGACAGACT	CGGGCTGCATGGGGAATGCGA
lpxD	GCCACGCTTGGCGTTTGCTGCT	CAGTTCGCGCCAATCACCACAT

Table S6. Extracellular Vesicles Buffer system

Reagent name	Molecular weight	Weigh the mass
2-Amino-2-(hydroxymethyl)-1,3-propanediol	121.14 g/mol	0.6057 g
NaCl	58.44 g/mol	0.0292 g
$MgSO_4$	120.37 g/mol	0.0120 g
DI water		to 100 mL

Adjust the pH to 7.4, store at 4°C for short-term storage, store at -20°C for long-term storage.

Text S1. DNA sequences used for constructing mutant strains

The sequence information used in this study are available in the NCBI GenBank database, gene accession numbers are as follows: *S.elongatus* PCC 7942 (CP130602.1), *luxCDABE* operon (OK165504.1), P_{psbAI} promoter (X04616.2), P_{kaiBC} promoter (CP130602.1), Kanamycin resistance cassette (AY048743.1), Spectinomycin resistance cassette (L05082.1), IPTG-inducible promoter (U85202.1), Gentamicin resistance cassette (AJ414668.1), and *rpaA* ORF (CP130602). pGEN-*luxCDABE* plasmid are available in http://n2t.net/addgene:44918. The gene sequences are as follows:

A. *luxCDABE* operon (synthetic construct) OK165504.1

ATGACTAAAAAAATTTCATTCATTATTAACGGCAGGTTGAAATCTTTCCCGAAGGTGAT

GATTTAGTGCAATCCATTAATTTTGGTGATAATAGTGTTTACCTGCCAATATTGAATGAC
TCTCATGTAAAAAACATTATTGATTGTAATGGAAATAACGAATTACGGTTGCATAACAT
TGTCAATTTTCTCTATACGGTAGGGGCAAAGATGGAAAAATGAAGAATACTCAAGACG
CAGGACATACATTCGTGACTTAAAAAAATATATGGGATATTCAGAAGAAATGGCTAAG
CTAGAGGCCAATTGGATATCTATGATTTTATGTTCTAAAGGCGGCCTTTATGATGTTGTA
GAAAATGAACTTGGTTCTCGCCATATCATGGATGAATGGCTACCTCAGGATGAAAGTT
ATGTTCGGGCTTTTCCGAAAGGTAATCTGTACATCTGTTGGCAGGTAATGTTCCATTATC
TGGGATCATGTCTATATTACGCGCAATTTTAACTAAGAATCAGTGTATTATAAAAACATC
GTCAACCGATCCTTTTACCGCTAATGCATTAGCGTTAAGTTTTATTGATGTAGACCCTAA
TCATCCGATAACGCGCTCTTTATCTGTTATATATTGGCCCCACCAAGGTGATACATCACT
CGCAAAAGAAATTATGCAACATGCGGATGTTATCGTCGCTTGGGGAGGGCCAGATGCG
ATTAATTGGGCGGTAGAGCATGCGCCATCTTATGCTGATGTGATTAAATTTGGTTCTAAA
AAGAGTCTTTGCATTATCGATAATCCTGTTGATTTGACGTCCGCAGCGACAGGTGCGGC
TCATGATGTTTGTTTTTACGATCAGCGAGCTTGTTTTTCTGCCCAAAACATATATTACAT
GGGAAATCATTATGAGGAATTTAAGTTAGCGTTGATAGAAAAACTTAATCTATATGCGC
ATATATTACCGAATGCCAAAAAAGATTTTGATGAAAAAGGCGGCCTATTCTTTAGTTCA
AAAAGAAAGCTTGTTTGCTGGATTAAAAGTAGAGGTGGATATTCATCAACGTTGGATG
ATTATTGAGTCAAATGCAGGTTGTGGAATTTAATCAACCACTTGGCAGATGTGTGTACC
TTCATCACGTCGATAATATTGAGCAAATATTGCCTTATGTTCAAAAAATAAAGACGCAA
ACCATATCTATTTTTCCTTGGGAGTCATCATTTAAATATCGAGATGCGTTAGCATTTAAA
GGTGCGGAAAGGATTGTAGAAGCAGGAATGAATAACATATTTCGAGTTGGTGGATCTC
ATGACGGAATGAGACCGTTGCAACGATTAGTGACATATATTTCTCATGAAAGGCCATCT
AACTATACGGCTAAGGATGTTGCGGTTGAAATAGAACAGACTCGATTCCTGGAAGAA
GATAAGTTCCTTGTATTTGTCCCATAATAGGTAAAAAGTATGGAAAATGAATCAAAATA
TAAAACCATCGACCACGTTATTTGTGTTGAAGGAAATAAAAAAATTCATGTTTGGGAA
ACGCTGCCAGAAGAAAACAGCCCAAAGAGAAAGAATGCCATTATTATTGCGTCTGGT
TTTGCCCGCAGGATGGATCATTTTGCTGGTCTGGCGGAATATTTATCGCGGAATGGATT
TCATGTGATCCGCTATGATTCGCTTCACCACGTTGGATTGAGTTCAGGGACAATTGATG
AATTTACAATGTCTATAGGAAAGCAGAGCTTGTTAGCAGTGGTTGATTGGTTAACTACA
CGAAAAATAAATAACTTCGGTATGTTGGCTTCAAGCTTATCTGCGCGGATAGCTTATGC
AAGCCTATCTGAAATCAATGCTTCGTTTTTTAATCACCGCAGTCGGTGTTGTTAACTTAA
GATATTCTCTTGAAAGAGCTTTAGGGTTTGATTATCTCTCAGTCTACCCATTAATGAATT
GCCGAATAATCTAGATTTTGAAGGCCATAAATTGGGGTGCTGAAGTCTTTGCGAGAGA
TTGTCTTGATTTTGGTTGGGAAGATTTAGCTTCTACAATTAATAACATGATGTATCTTGAT
ATACCGTTTATTGCTTTTACTGCAAATAACGATAATTGGGTCAAGCAAGATGAAGTTAT
CACATTGTTATCAAATATTCGTAGTAATCGATGCAAGATATATTTCTTTGTTAGGAAGTT
CGCATGACTTGAGTGAAAATTTAGTGGTCCTGCGCAATTTTTATCAATCGGTTACGAAA
GCCGCTATCGCGATGGATAATGATCATCTGGATATTGATGTTGATATTACTGAACCGTCA
TTTGAACATTTAACTATTGCGACAGTCAATGAACGCCGAATGAGAATTGAGATTGAAA
ATCAAGCAATTTCTCTGTCTTAAAATCTATTGAGATATTCTATCACTCAAATAGCAATAT
AAGGACTCTCTATGAAATTTGGAAACTTTTTGCTTACATACCAACCTCCCCAATTTTCTC
AAACAGAGGTAATGAAACGTTTGGTTAAATTAGGTCGCATCTCTGAGGAGTGTGGTTT
TGATACCGTATGGTTACTGGAGCATCATTTCACGGAGTTTGGTTTGCTTGGTAACCCTTA
TGTCGCTGCTGCATATTTACTTGGCGCGACTAAAAAATTGAATGTAGGAACTGCCGCTA

TTGTTCTTCCCACAGCCCATCCAGTACGCCAACTTGAAGATGTGAATTTATTGGATCAA
ATGTCAAAAGGACGATTTCGGTTTGGTATTTGCCGAGGGCTTTACAACAAGGACTTTC
GCGTATTCGGCACAGATATGAATAACAGTCGCGCCTTAGCGGAATGCTGGTACGGGCT
GATAAAGAATGGCATGACAGAGGGATATATGGAAGCTGATAATGAACATATCAAGTTC
CATAAGGTAAAAGTAAACCCCGCGGCGTATAGCAGAGGTGGCGCACCGGTTTATGTG
GTGGCTGAATCAGCTTCGACGACTGAGTGGGCTGCTCAATTTGGCCTACCGATGATATT
AAGTTGGATTATAAATACTAACGAAAAGAAAGCACAACTTGAGCTTTATAATGAAGTG
GCTCAAGAATATGGGCACGATATTCATAATATCGACCATTGCTTATCATATATAACATCT
GTAGATCATGACTCAATTAAAGCGAAAGAGATTTGCCGGAAATTTCTGGGGGCATTGG
TATGATTCTTATGTGAATGCTACGACTATTTTTGATGATTCAGACCAAACAAGAGGTTAT
GATTTCAATAAAGGGCAGTGGCGTGACTTTGTATTAAAAGGACATAAAGATACTAATC
GCCGTATTGATTACAGTTACGAAATCAATCCCGTGGGAACGCCGCAGGAATGTATTGA
CATAATTCAAAAAGACATTGATGCTACAGGAATATCAAATATTTGTTGTGGATTTGAAG
CTAATGGAACAGTAGACGAAATTATTGCTTCCATGAAGCTCTTCCAGTCTGATGTCATG
CCATTTCTTAAAGAAAAACAACGTTCGCTATTATATTAGCTAAGGAGAAAGAAATGAA
ATTTGGATTGTTCTTCCTTAACTTCATCAATTCAACAACTGTTCAAGAACAAAGTATAG
TTCGCATGCAGGAAATAACGGAGTATGTTGATAAGTTGAATTTTGAACAGATTTTAGTG
TATGAAAATCATTTTTCAGATAATGGTGTTGTCGGCGCTCCTCTGACTGTTTCTGGTTTT
CTGCTCGGTTTAACAGAGAAAATTAAAATTGGTTCATTAAATCACATCATTACAACTCA
TCATCCTGTCCGCATAGCGGAGGAAGCTTGCTTATTGGATCAGTTAAGTGAAGGGAGA
TTTATTTTAGGGTTTAGTGATTGCGAAAAAAAGATGAAATGCATTTTTTTAATCGCCCG
GTTGAATATCAACAGCAACTATTTGAAGAGTGTTATGAAATCATTAACGATGCTTTAAC
AACAGGCTATTGTAATCCAGATAACGATTTTTATAGCTTCCCTAAAATATCTGTAAATCC
CCATGCTTATACGCCAGGCGGACCTCGGAAAATATGTAACAGCAACCAGTCATCATAT
TGTTGAGTGGGCGGCCAAAAAAGGTATTCCTCTCATCTTTAAGTGGGATGATTCTAATG
ATGTTAGATATGAATATGCTGAAAGATATAAAGCCGTTGCGGATAAATATGACGTTGAC
CTATCAGAGATAGACCATCAGTTAATGATATTAGTTAACTATAACGAAGATAGTAATAA
AGCTAAACAAGAGACGCGTGCATTTATTAGTGATTATGTTCTTGAAATGCACCCTAATG
AAAATTTCGAAAATAAACTTGAAGAAATAATTGCAGAAAACGCTGTCGGAAATTATAC
GGAGTGTATAACTGCGGCTAAGTTGGCAATTGAAAAGTGTGGTGCGAAAAGTGTATTG
CTGTCCTTTGAACCAATGAATGATTTGATGAGCCAAAAAAATGTAATCAATATTGTTGA
TGATAATATTAAGAAGTACCACATGGAATATACCTAATAGATTTCGAGTTGCAGCGAGG
CGGCAAGTGAACGAATCCCCAGGAGCATAGATAACTATGTGACTGGGGTGAGTGAAA
GCAGCCAACAAAGCAGCAGCTTGAAAGATGAAGGGTATAAAAGAGTATGACAGCAG
TGCTGCCATACTTTCTAATATTATCTTGAGGAGTAAAACAGGTATGACTTCATATGTTGA
TAAACAAGAAATTACAGCAAGCTCAGAAATTGATGATTTGATTTTTCGAGCGATCCATT
AGTGTGGTCTTACGACGAGCAGGAAAAAATCAGAAAGAAACTTGTGCTTGATGCATT
TCGTAATCATTATAAACATTGTCGAGAATATCGTCACTACTGTCAGGCACACAAAGTAG
ATGACAATATTACGGAAATTGATGACATACCTGTATTCCCAACATCGGGTTTTAAGTTT
ACTCGCTTATTAACTTCTCAGGAAAACGAGATTGAAAGTTGGTTTACCAGTAGCGGCA
CGAATGGTTTAAAAAGTCAGGTGGCGCGTGACAGATTAAGTATTGAGAGACTCTTAGG
CTCTGTGAGTTATGGCATGAAATATGTTGGTAGTTGGTTTGATCATCAAATAGAATTAGT
CAATTTGGGACCAGATAGATTTAATGCTCATAATATTTGGTTTAAATATGTTATGAGTTT
GGTGGAATTGTTATATCCTACGACATTTACCGTAACAGAAGAACGAATAGATTTTGTTA

AAACATTGAATAGTCTTGAACGAATAAAAAATCAAGGGAAAGATCTTTGTCTTATTGG
TTCGCCATACTTTATTTATTTACTCTGCCATTATATGAAAGATAAAAAAATCTCATTTTCT
GGAGATAAAAGCCTTTATATCATAACCGGAGGCGGCTGGAAAAGTTACGAAAAAGAA
TCTCTGAAACGTGATGATTTCAATCATCTTTTATTTGATACTTTCAATCTCAGTGATATTA
GTCAGATCCGAGATATATTTAATCAAGTTGAACTCAACACTTGTTTCTTTGAGGATGAA
ATGCAGCGTAAACATGTTCCGCCGTGGGTATATGCGCGAGCGCTTGATCCTGAAACGT
TGAAACCTGTACCTGATGGAACGCCGGGGTTGATGAGTTATATGGATGCGTCAGCAAC
CAGTTATCCAGCATTTATTGTTACCGATGATGTCGGGATAATTAGCAGAGAATATGGTA
AGTATCCCGGCGTGCTCGTTGAAATTTTACGTCGCGTCAATACGAGGACGCAGAAAGG
GGTGTGCTTTAAGCTTAACCGAAGCGTTTGATAGTTGA

B. ***P**$_{kaiBC}$* **promoter CP130602.1**

TACCCGCCTACGAAAGCCAAAAGCTGCACCAAGCGATGCAGACTAGCTATCGTGAAA
TCGTTTTGAGCTATTTTTCGCCGAATAGCAACCTCAACCAGAGCATTGACAACTTCGTC
AACATGGCTTTCTTTGCCGATGTTCCAGTCACCAAAGTGGTAGAAATTCACATGGAGC
TGATGGACGAGTTTGCCAAGAAGCTCCGCGTAGAGGGACGTTCAGAGGACATTTTGC
TGGATTATCGGCTGACTTTAATTGATGTAATTGCACATCTTTGTGAGATGTATCGACGGT
CTATCCCACGAGAAACCTGAAAAGGTAAAGGAGGTCTTAAGCTCGGCTCAATTTCTCT
CTTTATCCTGTTAGATGGTTTGATTGCTGTTGCTACCCCGTTGATCTGCGT

C. **P**$_{psbAI}$ **promoter (psba-I gene)X04616.2**

CCCGCTGTTCTGGTGTCAGATTGCGCTCAAAGGACTCATGGCAGTCGCGAGCCTGCTG
CTCGTCGCAAGTCGCAATGCACGAGTAAAGAATGCCCGCCGGGTCGAATTGTTCATTT
ACCCAAATCACTTTGTCGGTTGCCATAGGGGGTTGCTCCTACGCTCAGCTGGATTTAGC
GTCTTCTAATCCAGTGTAGACAGTAGTTTTGGCTCCGTTGAGCACTGTAGCCTTGGGCG
ATCGCTCTAAACATTACATAAATTCACAAAGTTTTCGTTACATAAAAATAGTGTCTACTT
AGCTAAAAATTAAGGGTTTTTTACACCTTTTTGACAGTTAATCTCCTAGCCTAAAAAGC
AAGAGTTTTTAACTAAGACTCTTGCCCTTTACAACCTCAAGATCGAT

D. IPTG-inducible promoter (pGH/F2.79-242) U85202.1

GACACCATCGAATGGTGCAAAACCTTTCGCGGTATGGCATGATAGCGCCCGGAAGAG
AGTCAATTCAGGGTGGTGAATGTGAAACCAGTAACGTTATACGATGTCGCAGAGTATG
CCGGTGTCTCTTATCAGACCGTTTCCCGCGTGGTGAACCAGGCCAGCCACGTTTCTGC
GAAAACGCGGGAAAAAGTGGAAGCGGCGATGGCGGAGCTGAATTACATTCCCAACC
GCGTGGCACAACAACTGGCGGGCAAACAGTCGTTGCTGATTGGCGTTGCCACCTCCA
GTCTGGCCCTGCACGCGCCGTCGCAAATTGTCGCGGCGATTAAATCTCGCGCCGATCA
ACTGGGTGCCAGCGTGGTGGTGTCGATGGTAGAACGAAGCGGCGTCGAAGCCTGTAA
AGCGGCGGTGCACAATCTTCTCGCGCAACGCGTCAGTGGGCTGATCATTAACTATCCG
CTGGATGACCAGGATGCCATTGCTGTGGAAGCTGCCTGCACTAATGTTCCGGCGTTATT
TCTTGATGTCTCTGACCAGACACCCATCAACAGTATTATTTTCTCCCATGAAGACGGTA
CGCGACTGGGCGTGGAGCATCTGGTCGCATTGGGTCACCAGCAAATCGCGCTGTTAGC
GGGCCCATTAAGTTCTGTCTCGGCGCGTCTGCGTCTGGCTGGCTGGCATAAATATCTCA
CTCGCAATCAAATTCAGCCGATAGCGGAACGGGAAGGCGACTGGAGTGCCATGTCCG
GTTTTCAACAAACCATGCAAATGCTGAATGAGGGCATCGTTCCCACTGCGATGCTGGT
TGCCAACGATCAGATGGCGCTGGGCGCAATGCGCGCCATTACCGAGTCCGGGCTGCG
CGTTGGTGCGGATATCTCGGTAGTGGGATACGACGATACCGAAGACAGCTCATGTTAT
ATCCCGCCGTTAACCACCATCAAACAGGATTTTCGCCTGCTGGGGCAAACCAGCGTGG

ACCGCTTGCTGCAACTCTCTCAGGGCCAGGCGGTGAAGGGCAATCAGCTGTTGCCCG
TCTCACTGGTGAAAAGAAAAACCACCCTGGCGCCCAATACGCAAACCGCCTCTCCCC
GCGCGTTGGCCGATTCATTAATGCAGCTGGCACGACAGGTTTCCCGACTGGAAAGCG
GGCAGTGATTGACAATTAATCATCCGGCTCGTATAATGTGTGGAATTGTGAGCGGATAA
CAAAGAGG

E. *rpaA* ORF CP130602

ATGAAACCCCGCATCCTCGTGATCGATGATGACTCAGCCATCTTGGAGCTGGTCGCCG
TCAATCTGGAGATGTCTGGCTATGACGTACGCAAAGCTGAGGACGGCATTAAAGGTCA
GGCTTTAGCTGTTCAGCTAGTTCCCGACCTGATCATGCTGGATCTAATGCTGCCGCGGG
TTGATGGCTTTACCGTCTGTCAGCGACTGCGGCGCGATGAGCGTACTGCCGAAATTCC
GGTGCTGATGCTGACCGCCCTCGGACAGACTCAGGATAAGGTTGAAGGCTTCAACGC
GGGTGCTGACGATTATCTGACTAAGCCCTTCGAAGTTGAAGAGATGCTGGCCCGCGTG
CGTGCCTTGCTGCGGCGCACCGATCGCATTCCCCATGCAGCCCGCCATAGCGAAATTC
TCAGCTACGGTCCGCTGACCCTGATTCCCGAGCGGTTTGAGGCCATTTGGTTCAACCG
CACGGTCAAGCTGACTCACTTGGAATTTGAGTTGTTGCACTGCCTGTTGCAACGCCAC
GGCCAAACGGTTGCGCCGAGCGAAATCCTCAAAGAAGTCTGGGGCTATGATCCCGAC
GATGACATCGAGACGATTCGCGTCCACATCCGTCATCTGCGCACCAAGCTCGAGCCCG
ATCCCCGGCACCCGCGCTACATCAAAACGGTCTATGGAGCGGGCTACTGCCTTGAGCT
GCCGGCCGAGACGGAACTCCACCAACACGCCGATCAGTTTCCTTCGGCGTCCTGA

F. Kanamycin resistance cassette (pKD4) AY048743.1

ATTACACGTCTTGAGCGATTGTGTAGGCTGGAGCTGCTTCGAAGTTCCTATACTTTCTA
GAGAATAGGAACTTCGGAATAGGAACTTCAAGATCCCCTCACGCTGCCGCAAGCACT
CAGGGCGCAAGGGCTGCTAAAGGAAGCGGAACACGTAGAAAGCCAGTCCGCAGAA
ACGGTGCTGACCCCGGATGAATGTCAGCTACTGGGCTATCTGGACAAGGGAAAACGC
AAGCGCAAAGAGAAAGCAGGTAGCTTGCAGTGGGCTTACATGGCGATAGCTAGACTG
GGCGGTTTTATGGACAGCAAGCGAACCGGAATTGCCAGCTGGGGCGCCCTCTGGTAA
GGTTGGGAAGCCCTGCAAAGTAAACTGGATGGCTTTCTTGCCGCCAAGGATCTGATGG
CGCAGGGGATCAAGATCTGATCAAGAGACAGGATGAGGATCGTTTCGCATGATTGAA
CAAGATGGATTGCACGCAGGTTCTCCGGCCGCTTGGGTGGAGAGGCTATTCGGCTATG
ACTGGGCACAACAGACAATCGGCTGCTCTGATGCCGCCGTGTTCCGGCTGTCAGCGC
AGGGGCGCCCGGTTCTTTTTGTCAAGACCGACCTGTCCGGTGCCCTGAATGAACTGCA
GGACGAGGCAGCGCGGCTATCGTGGCTGGCCACGACGGGCGTTCCTTGCGCAGCTGT
GCTCGACGTTGTCACTGAAGCGGGAAGGGACTGGCTGCTATTGGGCGAAGTGCCGGG
GCAGGATCTCCTGTCATCTCACCTTGCTCCTGCCGAGAAAGTATCCATCATGGCTGATG
CAATGCGGCGGCTGCATACGCTTGATCCGGCTACCTGCCCATTCGACCACCAAGCGAA
ACATCGCATCGAGCGAGCACGTACTCGGATGGAAGCCGGTCTTGTCGATCAGGATGAT
CTGGACGAAGAGCATCAGGGGCTCGCGCCAGCCGAACTGTTCGCCAGGCTCAAGGC
GCGCATGCCCGACGGCGAGGATCTCGTCGTGACCCATGGCGATGCCTGCTTGCCGAAT
ATCATGGTGGAAAATGGCCGCTTTTCTGGATTCATCGACTGTGGCCGGCTGGGTGTGG
CGGACCGCTATCAGGACATAGCGTTGGCTACCCGTGATATTGCTGAAGAGCTTGGCGG
CGAATGGGCTGACCGCTTCCTCGTGCTTTACGGTATCGCCGCTCCCGATTCGCAGCGC
ATCGCCTTCTATCGCCTTCTTGACGAGTTCTTCTGAGCGGGACTCTGGGGTTCGAAATG
ACCGACCAAGCGACGCCCAACCTGCCATCACGAGATTTCGATTCCACCGCCGCCTTCT
ATGAAAGGTTGGGCTTCGGAATCGTTTTCCGGGACGCCGGCTGGATGATCCTCCAGCG

CGGGGATCTCATGCTGGAGTTCTTCGCCCACCCCAGCTTCAAAAGCGCTCTGAAGTTC
CTATACTTTCTAGAGAATAGGAACTTCGGAATAGGAACTAAGGAGGATATTCATAT

G. Spectinomycin resistance cassette(pRL277) L05082.1

CCATACAGAAGCTGGGCGAACAAACGATGCTCGCCTTCCAGAAAACCGAGGATGCG
AACCACTTCATCCGGGGTCAGCACCACCGGCAAGCGCCGCGACGGCCGAGGTCTTCC
GATCTCCTGAAGCCAGGGCAGATCCGTGCACAGCACCTTGCCGTAGAAGAACAGCAA
GGCCGCCAATGCCTGACGATGCGTGGAGACCGAAACCTTGCGCTCGTTCGCCAGCCA
GGACAGAAATGCCTCGACTTCGCTGCTGCCCAAGGTTGCCGGGTGACGCACACCGTG
GAAACGGATGAAGGCACGAACCCAGTGGACATAAGCCTGTTCGGTTCGTAAGCTGTA
ATGCAAGTAGCGTATGCGCTCACGCAACTGGTCCAGAACCTTGACCGAACGCAGCGG
TGGTAACGGCGCAGTGGCGGTTTTCATGGCTTGTTATGACTGTTTTTTTGGGGTACAGT
CTATGCCTCGGGCATCCAAGCAGCAAGCGCGTTACGCCGTGGGTCGATGTTTGATGTT
ATGGAGCAGCAACGATGTTACGCAGCAGGGCAGTCGCCCTAAAACAAAGTTAAACAT
CATGAGGGAAGCGGTGATCGCCGAAGTATCGACTCAACTATCAGAGGTAGTTGGCGT
CATCGAGCGCCATCTCGAACCGACGTTGCTGGCCGTACATTTGTACGGCTCCGCAGTG
GATGGCGGCCTGAAGCCACACAGTGATATTGATTTGCTGGTTACGGTGACCGTAAGGC
TTGATGAAACAACGCGGCGAGCTTTGATCAACGACCTTTTGGAAACTTCGGCTTCCCC
TGGAGAGAGCGAGATTCTCCGCGCTGTAGAAGTCACCATTGTTGTGCACGACGACATC
ATTCCGTGGCGTTATCCAGCTAAGCGCGAACTGCAATTTGGAGAATGGCAGCGCAATG
ACATTCTTGCAGGTATCTTCGAGCCAGCCACGATCGACATTGATCTGGCTATCTTGCTG
ACAAAAGCAAGAGAACATAGCGTTGCCTTGGTAGGTCCAGCGGCGGAGGAACTCTTT
GATCCGGTTCCTGAACAGGATCTATTTGAGGCGCTAAATGAAACCTTAACGCTATGGA
ACTCGCCGCCCGACTGGGCTGGCGATGAGCGAAATGTAGTGCTTACGTTGTCCCGCAT
TTGGTACAGCGCAGTAACCGGCAAAATCGCGCCGAAGGATGTCGCTGCCGACTGGGC
AATGGAGCGCCTGCCGGCCCAGTATCAGCCCGTCATACTTGAAGCTAGACAGGCTTAT
CTTGGACAAGAAGAAGATCGCTTGGCCTCGCGCGCAGATCAGTTGGAAGAATTTGTC
CACTACGTGAAAGGCGAGATCACCAAGGTAGTCGGCAAATAATGTCTAACAATTCGTT
CAAGCCGACGCCGCTTCGCGGCGCGGCTTAACTCAAGCGTTAGATGCACTAAGCACA
TAATTGCTCACAGCCAAACTATCAGGTCAAGTCTGCTTTTATTATTTTTAAGCGTGCATA
ATAAGCCCTACACAAATTGGGAGATATATCATGAAAGGCTGGCTTTTTCTTGTTATCGC
AATAGTTGGCGAAGTAATCGCAACATCCGCATTAAAATCTAGCGAGGGCTTTACTAAG
CTGATCCGGTGGATGACCTTTTGAATGACCTTTAATAGATTATATTACTAATTAATTGGG
GACCCTAGA

H. Gentamicin resistance cassette(pCom11), AJ414668.1

CGATCTCGGCTTGAACGAATTGTTAGGTGGCGGTACTTGGGTCGATATCAAAGTGCATC
ACTTCTTCCCGTATGCCCAACTTTGTATAGAGAGCCACTGCGGGATCGTCACCGTAATC
TGCTTGCACGTAGATCACATAAGCACCAAGCGCGTTGGCCTCATGCTTGAGGAGATTG
ATGAGCGCGGTGGCAATGCCCTGCCTCCGGTGCTCGCCGGAGACTGCGAGATCATAG
ATATAGATCTCACTACGCGGCTGCTCAAACCTGGGCAGAACGTAAGCCGCGAGAGCG
CCAACAACCGCTTCTTGGTCGAAGGCAGCAAGCGCGATGAATGTCTTACTACGGAGC
AAGTTCCCGAGGTAATCGGAGTCCGGCTGATGTTGGGAGTAGGTGGCTACGTCTCCGA
ACTCACGACCGAAAAGATCAAGAGCAGCCCGCATGGATTTGACTTGGTCAGGGCCGA
GCCTACATGTGCGAATGATGCCCATACTTGAGCCACCTAACTTTGTTTTAGGGCGACTG
CCCTGCTGCGTAACATCGTTGCTGCTGCGTAACATCGTTGCTGCTCCATAACATCAAAC

ATCGACCCACGGCGTAACGCGCTTGCTGCTTGGATGCCCGAGGCATAGACTGTACAAA
AAAACAGTCATAACAAGCCATGAAAACCGCCACTGCGCCGTTACCACCGCTGCGTTC
GGTCAAGGTTCTGGACCAGTTGCGTGAGCGCATACGCTACTTGCATTACAGTTTACGA
ACCGAACAGGCTTATGTCAATTCGCCTCTCAGGCGCCGCTGGTGCCGCTGGTTGGACG
CCAAGGGTGAATCCGCCTCGATACCCTGATTACTCGCTTCCTGCGCCCTCTCAGGCGG
CGATAGGGGACTGGTAAAACGGGGATTGCCCAGACGCCTCCCCCGCCCCTTCAGGGG
CACAAATGCGGCCCCAACGGGGCCACGTAGTGGTGCGTTTTTTGCGTTTCCACCCTTTT
CTTCCTTTTCCCTTTTAAACCTTTTAGGACGTCTACAGGCCACGTAATCCGTGGCCTGTA
GAGTTTAAAAAGGGACGGATTTGTTGCCATTAAGGGACGGATTTGTTGTTAAGAAGGG
ACGGATTTGTTGTTGTAAAGGGACGGATTTGTTGTATTGTGGGACGCAGATACAGTGTC
CCCTTATACACAAGGA
